# Behind the Doors of Mental Health Units: Staff Perspectives on Psychological Safety

**DOI:** 10.1192/j.eurpsy.2026.11976

**Published:** 2026-07-31

**Authors:** J. Q. L. Ong

**Affiliations:** Psychiatry, Institute of Mental Health, Singapore, Singapore

## Abstract

**Introduction:**

Psychological safety—defined as the ability to take interpersonal risks or admit mistakes without fear of negative consequences—is vital for staff well-being, teamwork, and care quality, yet under-researched in mental healthcare.

**Objectives:**

This study explores self-reported psychological safety among healthcare staff in acute inpatient mental health units in Singapore.

**Methods:**

A 21-item Likert-scale questionnaire (Vogt et al. Int J Qual Health Care 2024; 36:mzae086) was used, where agreement indicated positive perceptions. 34 staff from specialised inpatient mental health units in Singapore were surveyed. Additional questions assessed awareness and use of organisational mental health support services, with qualitative responses collected from those who felt they needed support but did not access it.

**Results:**

Overall, staff reported a positive perception of psychological safety, with strong team dynamics, though gaps remain in leadership communication and mental health support access.

**Team-Level Safety and Inclusivity:** 85.3% felt their team was inclusive of all backgrounds. 70.6% felt supported and respected by team members. 67.7% felt listened to and comfortable raising difficult issues. Only 20.6% had trouble asking for help. However, just 55.9% felt able to challenge others’ disrespectful behaviour towards patients, and 64.7% felt able to discuss risk openly—highlighting areas for improvement in communication and empowerment.

**Organisational Leadership and Culture:** 76.5% agreed their organisation values cultural diversity. 85.3% felt supported by their direct manager, but only 64.7% felt listened to by senior management and 58.8% felt valued by them—suggesting a disconnect between leadership and frontline staff. 64.7% believed they could raise concerns without fear of penalty, indicating moderate organisational psychological safety.

**Physical Safety and Preparedness:** 79.4% felt physical safety was prioritised, and 70% felt adequately trained. However, only 64.7% felt comfortable raising safety concerns about service users, pointing to stigma or fear that may inhibit openness around risk.

**Mental Health Support:** While 38.2% reported needing mental health support, only 8.8% accessed services. Barriers included lack of time, confidentiality concerns, and previous negative experiences with counselling. These findings reveal a substantial unmet need for accessible, trusted psychological support.

**Relationships with Service Users:** 70.6% felt able to develop meaningful relationships with service users—an essential element of therapeutic care.

**Conclusions:**

While results suggest an overall healthy team climate and commitment to inclusion and safety, key areas for development include improving senior leadership engagement, staff empowerment to raise concerns, and addressing barriers to mental health service access to ensure staff well-being and safer care environments.

**Disclosure of Interest:**

None Declared

